# Studying safe storage time of orange peel (Citrus reticulata) using high‐throughput sequencing and conventional pure culture

**DOI:** 10.1002/fsn3.866

**Published:** 2018-10-29

**Authors:** Fu Wang, Lin Chen, Sujuan Liu, Fengqing Li, Xin Zhang, Hongping Chen, Youping Liu

**Affiliations:** ^1^ Department of Pharmacy Chengdu University of TCM Standardization education ministry key laboratory of traditional Chinese medicine Chengdu Sichuan China; ^2^ Food & Drugs Authority of Nanchong Nanchong Sichuan China

**Keywords:** *Aspergillus* fungal, high‐throughput sequencing, orange peel, safe storage time

## Abstract

*Aspergillus* is a fungal genus widely studied all over the world because some species are known allergens and opportunistic human pathogens. The dynamic growth of *Aspergillus* is a prerequisite for establishing safe storage time of orange peel. In this paper, high‐throughput sequencing technique was used for the first time to analyze the diversity and structure of fungi in the same batch of samples at different periods of time, and 20 batches of fresh orange peel and 56 batches of dried peel were verified. Results shown that the orange peel gradually began to grow *Aspergillus* fungal after storing for 240 days, and the abundance became maximum at 270 days and then decreased. These results suggest the safe storage time should be from January to August. And orange peel should be dried in August to prevent rapid propagation or metabolic toxicity production of *Aspergillus* fungi.

## 1 INTRODUCTION

*Citrus reticulata* Blanco is widely cultivated in the world. The orange peel can be processed as food, tea drinks, seasoning, or even herb‐medicine (Rachma, Huong, Ria, Claes, & Mohammad, 2014; Zheng, Zeng, Peng, Wu, & Su, 2018), but it is easy to mildew during storage and infect pathogenic fungi or toxins, thus endangering the health of consumers. However, the microbial community structure, including composition, abundance, and diversity, is not fully understood during storage of orange peel, which we cannot put forward the safe storage time and scientific safe storage measures for orange peel.

Traditionally, pure culture methods have been used to identify pathogenic fungi. In these methods, isolation, identification, and biological characteristics of pathogenic fungi were studied (Yan, Chen, & Deng, 2007; Yang et al., 2015). However, these conventional methods are limited in identifying fungal communities in full scale. In addition, there are many kinds of fungi in the environment, and their growth, physiological, and biochemical characteristics will be unstable with the change of environment. So, the fungal community in the sample will also change with the storage time. Thus, a new detection method should be adopted in order to get a better understanding of the growth of fungi in orange peel.

In recent years, internal transcribed spacer (ITS) has been widely used in fungal taxonomic identification (Bachy, Dolan, López‐García, Deschamps, & Moreira, 2013; Bengtsson‐Palme, Ryberg, Hartmann, Branco, & Wang, 2013; Findley et al., 2013). Using HiSeq platform to sequence ITS1 region, it has the characteristics of high sequencing depth and low cost for identification of low abundance community species (Caporaso et al., 2012; Degnan & Ochman, 2012). High‐throughput sequencing can not only obtain the composition of microbial communities in the samples, but also digitize their relative contents, which provides a reliable and effective method for the analysis of the diversity of microbial communities in environmental samples. At present, high‐throughput sequencing technology has been widely applied in microbial diversity analysis in soil (Shen et al., 2015; Xia et al., 2011), human skin (Findley et al., 2013), grape surface (Bokulich, Thorngate, Richardson, & Mills, 2014), and other samples. The main techniques of microbial community diversity research include amplifier sequencing and macro genome sequencing. Amplifier sequencing is mainly carried out by sequencing PCR products of specific length. Its amplifier sequencing for environmental samples is one of the important techniques to study the diversity and community composition of environmental microorganisms.

In the present study, its amplifier sequencing technique was used to analyze the fungal diversity and community structure of 14 batches of orange peel (fresh orange peel, dried orange peel for 7 days/30 days / 60 days / 90 days / 120 days / 150 days / 180 days / 240 days / 270 days / 300 days / 330 days / 360 days). At the same time, 20 batches of fresh orange peel and 56 batches of aged peel were isolated and identified by conventional pure culture method. The objective of this study was to understand the growth dynamics and dominant flora of fungi in orange peel during storage and to predict the safe storage time as well as to provide scientific safe storage measures for orange peel.

## 2 METHOD AND MATERIALS

### 2.1 Materials

**Figure 1:**
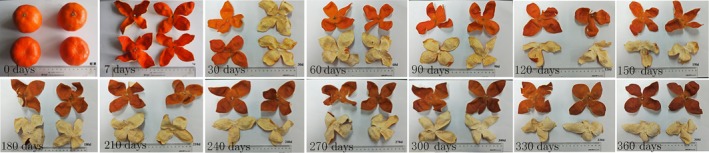
The samples used in the high‐throughput sequencing

In order to ensure the identity and individual difference of the orange peel, we collected the samples by means of fixed point, fixed plant, and fixed variety. The variety of Dahongpao (C. *reticulata*, “Dahongpao”) from Wugui Village, Qingquan Town, Qingjiang District, Chengdu City, Sichuan Province was selected (Figure 1).

### 2.2 Instruments and reagents

Retsch MM400 Ball Mill (Laichi co., Berlin, Germany); PTC200PCR (Bio‐Rad, Berkeley, CA, USA); GelDox XR gel imaging system (Bio‐Rad, USA); DYY‐8C Electrophoretic apparatus (Beijing Liuyi Instrument, China); BI3730XL sequenator (Applied Biosystems, Foster City, CA, USA); KRQ‐300P manual climatic box (ChongQing YinHe Test Instrument, China); Plant DNA Extraction Kit (TIANGEN Biotech Co., Chengdu, China); 2 × Taq PCR Master Mix (TIANGEN Biotech Co.,); primer was compounded by Sangon Co., China. Shimadzu; DM4000M microscope (Leica, Frankfurt, Germany); TruSeq^®^ DNA PCR‐Free Sample Preparation Kit (Illumina Co., San Diego, CA, USA); QIAquick gum recovery kit (QIAGEN, Duesseldorf, Germany); Phusion^®^ High‐Fidelity PCR Master Mix with GC Buffer (New England Biolabs, USA); High‐efficient enzyme (New England Biolabs, Boston, MA, USA).

### 2.3 High‐throughput sequencing method

By using sterile cotton swabs to wipe on the surface of orange peel, 20 cotton swabs were used for wiping area of 5 cm × 5 cm. After wiping, the cotton swabs were cut off from their poles with scissors. The genomic DNA was extracted by CTAB method, the purity and concentration of DNA were detected by agarose gel electrophoresis, the appropriate amount of sample was taken into centrifuge tube, and the sample was diluted to 10^‐6^ g L^−1^ with aseptic water. In order to ensure the efficiency and accuracy of amplification, the diluted genomic DNA was used as template, and the specific primer with Barcode, Phusion High‐Fidelity PCR Master Mix with GC Buffer, and high‐efficiency enzyme were used to carry out PCR. Primer corresponding region was ITS5‐1737F and ITS2‐2043R. PCR reaction system (30 μl): Phusion Master Mix (2×) 15 μl, positive and reverse primer (2 μmol/L) 1.5 μl each, DNA(1 mg/L) 10 μl, H_2_O 2 μl. PCR reaction procedure: 98°C 1 min; 98°C 10 s; 50°C 30 s; 72°C 30 s; 30 cycles; 72°C 5 min. PCR products were detected by using 2% agarose gel electrophoresis. According to the concentration of PCR products, the samples were mixed in the same amount, and then the PCR products were detected by 2% agarose gel electrophoresis, and the target bands were recovered by gel. TruSeq DNA PCR‐Free Sample Preparation Kit was used for library construction. The library was quantified by Qubit and qPCR. After the library was qualified, HiSeq 2500 PE250 was used for sequencing.

### 2.4 Statistical Analysis

According to the Barcode sequence and PCR amplified primer sequence, each sample datum was separated from the off computer data, and the reads of each sample were spliced by flash v1.2.7 (Magč & Salzberg, 2011) after the Barcode and primer sequences were cut off, and the sequence obtained was raw tags data, which was filtered to obtain high‐quality tags data (Bokulich et al., 2013). Referring to QIIME v1.7.0 (Caporaso et al., 2010) for quality control of tags, the processed tag sequence was compared with the database (UNITE database) to detect chimeric sequences through UCHIME algorithm (Edgar, Haas, Clemente, Quince, & Knight, 2011), and the final effective tags were obtained by removing the chimeric sequence (Haas et al., 2011). By using UPARSE v7.0.1001 (Edgar, 2013), all effective tags of all samples were clustered, the sequences were clustered into OTUs (operational taxonomic units), and select representative sequences of OTUs) with 97% consistency, the sequences with the highest frequency in OTUs were selected as the representative sequences of OTUs. Using BLAST method in QIIME software (Altschul, Gish, Miller, Myers, & Lipman, 1990) and UNIT database (Kõljalg, Nilsson, & Abarenkov, 2013), the species annotation analysis of the representative sequence of OTUs was carried out, and the community composition of each sample was analyzed at each classification level. The data of each sample were homogenized according to the minimum amount of data in the sample, and the subsequent alpha diversity analysis and beta diversity analysis were based on the data after homogenization. The QIIME software was used to calculate the ACE and Good‐coverage index of the Shannon Simpson, and the unweighted pair‐group method was used to construct the cluster tree of UPGMA samples.

### 2.5 Microscopic Identification of growing Fungi in Orange Peel

Methods for isolation of fungi in orange peel with reference to the methods reported in the literature (Jiang, Qian, & Duan, 2008) and modified as follows: Take 5.0 g for each batch of PCR, put in the 50‐ml sterile centrifuge tube, add in 30 ml 0.1% Tween‐20, agitate violently in 3 min, filter with disposable syringe, collect filtrate, 5000 rpm centrifuge in 10 min, discard the supernatant, resuspend the sediment in 300 μl 40% sterile glycerinum, dilute to 1 × 10^−4^ bacterium liquid, respectively, take 100 μl to coat on PDA plate (chloromycetin concentration 0.1 g L^−1^), and incubate 3 days in constant temperature 30°C. After observation of medium grow colonies, transfer to the new PDA plate by inoculating loop (chloromycetin concentration 0.1 g L^−1^), continue to cultivate, inoculate and transfer to PDA test tube, and then strain identification was carried out. In the ultraclean workbench, put the coverslip on PDA medium inclined, inoculate the purified strains with inoculating loop, and cultivate 3 days in the incubator in constant temperature 30°C. Shave the medium lower end of the coverslip by blade, and drip a drop of distilled water on the center of the glass slide. Then put on the coverslip, observe and take pictures by low‐power lens and high‐power lens under a microscope.

### 2.6 Molecular identification of growth fungi in orange peel

Extract DNA from samples by DNA extraction kits; amplify ITS sequence by universal primer ITS1/ITS4 of DNA barcode. Reaction conditions and amplification procedure were described by Hou, Song, Yang, Zhou, and Yao (2014). Bidirectional DNA sequence after PCR products has purified. Proofread and splice the sequence map which comes from data processing by soft CodonCodeAligner V5.0.2, remove the primer and inferior quality sequence. Construct neighbor‐joining phylogenetic tree by soft Maga 5.1, check the approval rating of each branch by Bootstrap. Identify and analyze by similarity search method.

## 3 RESULTS AND DISCUSSION

### 3.1 High‐throughput sequencing of growth Fungi from Orange Peel

By using Illumina HiSeq sequencing platform, raw PE data were obtained and quality control was carried out; clean tags were obtained and then chimeric filtering was carried out; and effective data, which could be used for subsequent analysis, were obtained. The effective tags were clustered, and the sequences were clustered into OTUs (operational taxonomic units) with 97% identity, and then, the representative sequences of OTUs were annotated. As shown in Table 1, the total number of samples available at each sampling point was about 47679, with a range of 471–975.

**Table 1:** The effective sequence of sampling points and the quantitative statistics of OTU

No.	Effective tags	Base(nt)	AvgLen(nt)	OTU quantity
cx	78159	20376423	263	471
7 d	81125	22606578	279	749
30 d	74590	18483412	249	719
60 d	80233	19195254	239	685
90 d	79701	17955550	226	975
120 d	75148	18236216	242	605
150 d	67549	17799910	270	534
180 d	68863	16679672	241	789
210 d	74481	17115153	230	730
240 d	79981	19706548	246	880
270 d	90125	19815669	220	813
300 d	84629	18834326	223	750
330 d	74227	15494247	211	564
360 d	81178	15534979	191	515

Effective tags are sequences that filter chimerism and are ultimately used for subsequent analysis. Base refers to the number of bases of the final effective data. AvgLen refers to the average length of effective tags.

D, days.

Alpha Diversity can reflect the microbial community diversity of a sample (Li, Zhang, Guo, Wu, & Zhang, 2013). The richness and diversity of microbial communities in the sample can be reflected by single sample diversity analysis (alpha diversity). The index of coverage refers to the sequence coverage of each sample, and the higher the value of coverage, the greater the probability of sequence detection in the sample, and the smaller the probability of not being detected, which directly represents the authenticity of the sequencing results of the sample (Kraková, Šoltys, Budiš, Pangallo, & Szemes, 2016). The results of Table 2 show that the coverage of all samples was above 0.994, which indicates that the coverage of fungi in the samples was high and the depth of sequencing was suitable, which could meet the needs of fungal diversity analysis in the samples (Qu, Zhang, Ma, Liu, & Ya, 2017).

**Table 2:** Diversity of bacterial community in different groups

Group	Shannon	Simpson	Chao1	ACE	Goods_coverage
CX	3.03	0.66	478	489	0.998
C7 d	3.23	0.64	805	844	0.996
C30 d	4.97	0.92	685	697	0.997
C60 d	5.04	0.92	741	759	0.997
C90 d	4.96	0.86	1095	1114	0.994
C120 d	4.45	0.85	624	638	0.997
C150 d	3.83	0.77	553	580	0.997
C180 d	4.96	0.87	836	839	0.996
C210 d	4.62	0.87	751	776	0.996
C240 d	4.59	0.86	980	1007	0.995
C270 d	4.74	0.89	921	949	0.995
C300 d	4.03	0.79	769	795	0.996
C330 d	3.45	0.69	601	625	0.997
C360 d	2.76	0.61	582	600	0.997

D, days.

According to the number of OTU and alpha diversity index, the variation of microbial species and abundance of orange peel from fresh peel to dry peel were significant, among them, there were few microbes in fresh peel, and with the prolongation of storage time, the temperature and humidity changed in storage, and the structure of microbial community changed significantly. After 90 days of storage, the species of microbes increased sharply and the abundance increased, the period of storage was also in spring, and the temperature and humidity gradually increased, and the microbial reproduction increased, which led to the increase of infected microorganism on the surface of orange peel, and when stored for 270 days or so, the species and abundance of microorganism decreased, this stage was the beginning of winter, the temperature decreased and the metabolism of microorganism was slow. It can be seen that the change of microbial community structure in orange peel was closely related to environmental factors.

**Figure 2:**
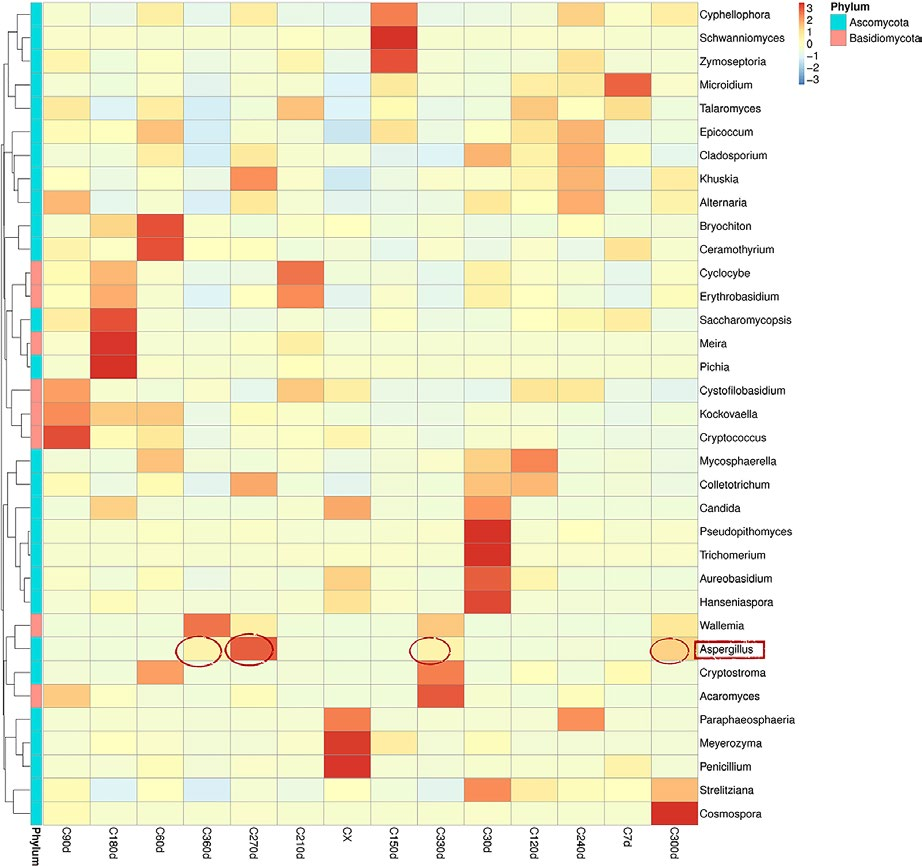
Plotted by sample name on the *x*‐axis and the *y*‐axis represents the genus. The cluster tree on the left side of the graph is a species cluster tree. Figure 2 Species abundance cluster map

**Figure 3:**
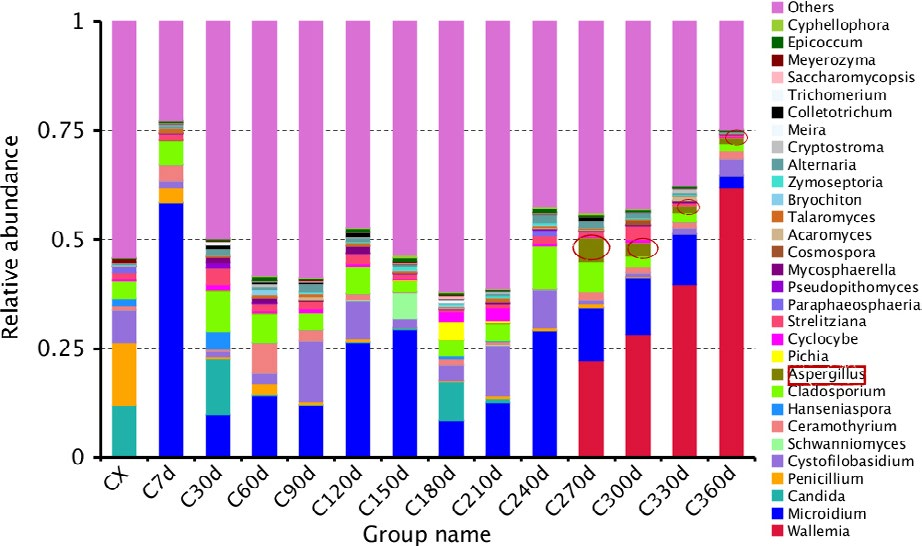
The abscissa is the sample name; the longitudinal coordinates indicate relative abundance. Figure 3 Relative abundance columnar graphs of species at the level of the genus

According to the species annotation and abundance information of all samples at the generic level (Figure 2), select the genus with the top 35 abundance, cluster the species and samples according to their abundance information in each sample, and draw the heat map (Figure 3). On the Heatmap, the species with high abundance and low abundance were grouped together, and the similarity and difference of the composition of multiple sample communities were reflected by the color gradient and similarity degree (Li et al., 2016). Define the species of Z > 1 in the Heatmap map as the dominant genus (Table 3). *Penicillium* and *Meyerozyma* gathered more in the fresh peel, and *Aspergillus* did not distribute in the early storage period (0 days–240 days) and gradually appeared in the late storage period (270 days–360 days), while the genus *Aureobasidium*,* Trichomerium*, and *Pseudopithomyces* mainly distributed in 30 days after storage. *Ceramothyrium* and *Bryochiton* gathered more when orange peel was stored for 60 days. *Cryptococcus* mainly existed in 90 days and *Schwanniomyces* in 150 days. *Pichia, Meira*, and *Saccharomycopsis* were found in 180 days, while *Acaromyces* was concentrated at 330 days, and *Wallemia* was abundant at 360 days.

**Table 3:** Distribution of dominant bacteria in orange peel in the process of “fresh, dry, and aged”

NO.	Dominant bacteria
CX	*Penicillium, Meyerozyma, Paraphaeosphaeriae, Candida*
C7 d	*Microidium*
C30 d	*Strelitziana, Hanseniaspora, Aureobasidium, Trichomerium, Pseudopithomyces, Candida, Colletotrichum, Cladosporium*
C60 d	*Kockovaella, Cryptostroma, Ceramothyrium, Epicoccum, Bryochiton*
C90 d	*Cryptococcus, Kockovaella, Cystofilobasidium, Alternaria*
C120 d	*Colletotrichum, Mycosphaerella, Talaromyces*
C150 d	*Zymoseptoria, Cyphellophora, Schwanniomyces*
C180 d	*Kockovaella, Pichia, Meira, Saccharomycopsis, Erythrobasidium, Cyclocybe*
C210 d	*Erythrobasidium, Cyclocybe, Talaromyces*
C240 d	*Paraphaeosphaeria, Alternaria, Khuskia, Epicoccum, Cladosporium*
C270 d	*Aspergillus, Colletotrichum, Khuskia*
C300 d	*Cosmospora*
C330 d	*Acaromyces, Cryptostroma*
C360 d	*Wallemia*

D, days.

The results of high‐throughput sequencing showed that *Penicillium* and *Meyerozyma* accumulated more in the fresh peel, while *Aspergillus* appeared in the 270‐day to 360‐day process at the late storage stage, but was not distributed in the early stage. It can be concluded that fresh orange peel does not contain *Aspergillus* fungi, but is infected by environment during storage.

Microbes are everywhere, and food will always be affected by microbes in the air. *Aspergillus* is a genus of fungi with more than 300 species reported in the literature, among which five and 51 species are known human allergens and opportunistic pathogens, respectively. Improper means of processing, storage, and transportation of food may lead to contamination of pathogenic bacteria. Rice, coix seed, corn, peach kernel, and so on are liable to be infected with *Aspergillus flavus* or aflatoxin. The existing standard is not perfect enough only to limit aflatoxin in food. The source of pathogenic or toxin‐producing fungal should be explored and establishes a safe storage time, so as to ensure the safety of consumers.

### 3.2 Microscopic and Molecular Identification of growing Fungi in Orange Peel

**Figure 4:**
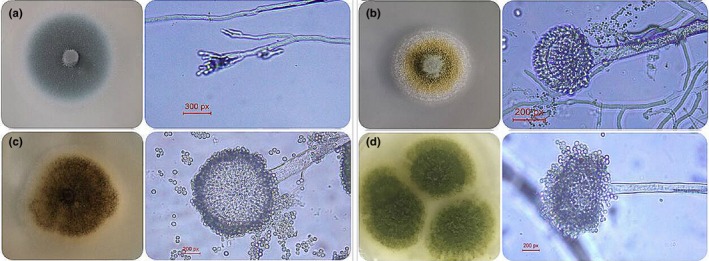
Morphology and micrograph of fungi. (*a*) *Penicillium citrinum;* (*b*) *Aspergillus flavus;* (*c*) *Aspergillus niger ;* (*d*) *Penicillium minioluteum*

In order to verify that orange peel is easy to be contaminated by *Aspergillus* from environment during storage, 20 batches of fresh orange peel and 56 batches of aged peel samples were isolated and identified by microscopical method. A total of 125 strains were isolated in total. Among them, 42 strains of fungi were isolated from fresh orange peel and 83 strains from aged peel. They were *Penicillium communne*,* Penicillium citrinum*,* Aspergillus flavus*,* Aspergillus niger*, and *Penicillium minioluteum*, which belong to two genera and five species. The morphology and micrograph of some strains are shown in Figure 4. There were no *Aspergillus* fungi in fresh orange peel, but two strains of *Aspergillus flavus* were found in the sample of aged peel.

**Figure 5:**
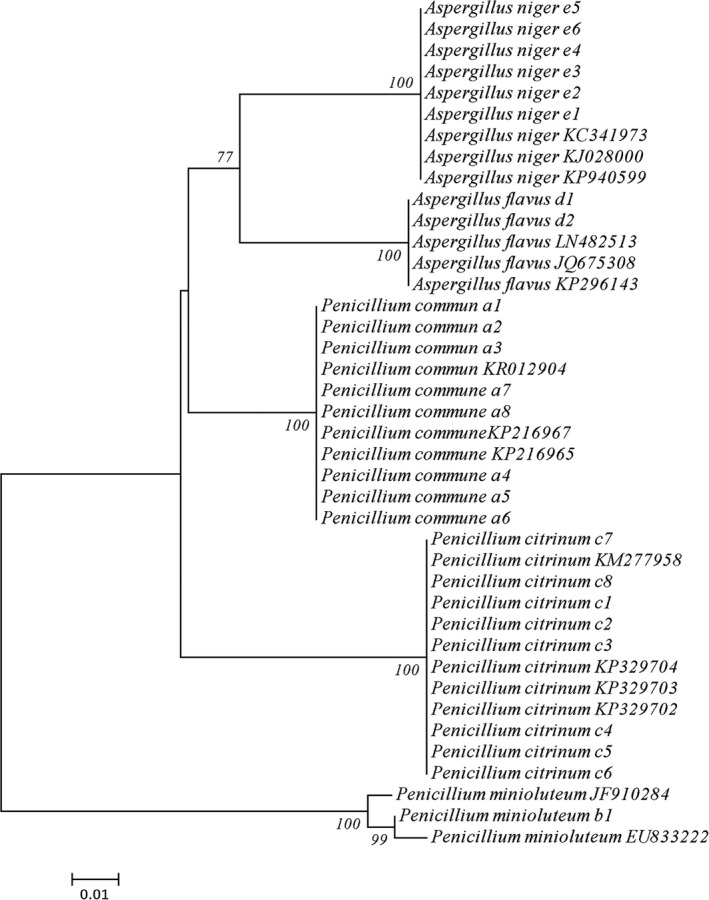
The NJ tree that constructed a base on the K2P distance of the ITS sequence

In order to identify the isolated strains more accurately, 25 strains of fungi were selected for DNA extraction, PCR amplification, and sequencing experiments. All the strains were amplified by PCR and sequenced successfully. The results of BLAST search showed that all the 25 strains belong to *Penicillium* or *Aspergillus*. At the same time, the phylogenetic tree of neighbor‐joining (Figure 5) could be used to distinguish different fungi, *Penicillium communne*,* Penicillium citrinum*,* Aspergillus flavus*,* Aspergillus niger*, and *Penicillium minioluteum* together with the corresponding NCBI download sequence.

The results further proved that the fresh orange peel did not contain *Aspergillus* fungi, while the aged peel contained pathogenic fungi such as *Aspergillus flavus*. It was suggested that orange peel can be safely used as food, tea drinks, or seasoning, and the safe storage time should not exceed 240 days after drying, that is, no longer than the August of the year. Moreover, in July, August, and September, when microbial activities were frequent, orange peel should be kept dry to avoid rapid propagation and metabolism of *Aspergillus* fungi. In our study, two strains of *Aspergillus flavus* were isolated from 56 batches of aged peel, and because of *Aspergillus flavus* can produce aflatoxin (Chen, Zhang, Yang, & Jin, 2005; Trucksess, Dombrink‐kurtman, Tournas, & White, 2002), there is a certain safety risk to use as food or herb‐medicine. And with the increase of storage time, the risk of infection with *Aspergillus* fungi in orange peel increased. Therefore, it is necessity to limit the safe storage time of orange peel due to human health impacts of *Aspergillus* fungi.

## 4 CONCLUSION

*Aspergillus* is a fungal genus widely studied due to human health impacts. In the current study, the safe storage time of orange peel was studied using high‐throughput sequencing and conventional pure culture for the first time. Results showed that *Aspergillus* fungal did not distribute in the early storage period (0 day–240 days) of orange peel and gradually appeared in the late storage period (270 days–360 days). It was suggested that fresh orange peel can be safely used as food, tea drinks, or seasoning, and the safe storage time of dried orange peel should not exceed 240 days after storing, that is, no longer than the August of the year. Moreover, in July, August, and September, when microbial activities were frequent, dried orange peel should be kept dry to avoid rapid propagation and metabolism of *Aspergillus* fungi. Our study provides not only a basic study for a safe storage time of orange peel, but also a reference for the safe storage of herb‐medicine which is susceptible to aflatoxin infection.

## CONFLICT OF INTEREST

No conflict of interest declared.

## ETHICAL STATEMENT

This study does not involve any human or animal testing.
